# The Role of Endogenous Hormones in Regulating Early Development of Stone Fruit

**DOI:** 10.3390/plants15060890

**Published:** 2026-03-13

**Authors:** Shuning Zhang, Yali Sun, Xiaofeng Zhou, Zhiwei Deng

**Affiliations:** 1 College of Horticulture and Forestry, Tarim University, Alar 843300, China; 2 National-Local Joint Engineering Laboratory of High Efficiency and Superior-Quality Cultivation and Fruit Deep Processing Technology on Characteristic Fruit Trees, Alar 843300, China; 3 Xinjiang Academy of Forestry Sciences, Urumqi 830062, China

**Keywords:** endogenous hormones, stone fruits, fruit early development

## Abstract

Stone fruits, mainly represented by *Prunus* species, are economically important crops whose yield potential and final quality are largely determined during early fruit development. This early phase, encompassing pollination, fertilization, fruit set, cell division, and pit hardening, involves irreversible developmental decisions that govern fruit survival, size, and productivity. In this review, recent advances in endogenous hormonal regulation during early stone fruit development are synthesized, with emphasis on auxin, gibberellin (GA), cytokinin (CTK), and abscisic acid (ABA). Auxin and GA act as core growth-promoting signals that synergistically initiate fruit set, stimulate cell division and expansion, and support parthenocarpy development, while CTK reinforces early cell proliferation and contributes to final fruit size. In contrast, ABA primarily functions as a growth-inhibitory regulator, integrating developmental and environmental cues to promote fruit growth arrest and abscission under unfavorable conditions. These hormones interact through dynamic synergistic and antagonistic networks that are continuously reprogrammed across developmental stages and tissues. This review provides a regulatory framework for understanding hormone-mediated early fruit development in stone fruits and offers guidance for orchard management and future molecular breeding to stabilize fruit set and improve yield and quality.

## 1. Introduction

Stone fruit trees, characterized by their hard and lignified endocarp, constitute a crucial category of economic crops. Primarily represented by members of the genus *Prunus* L. within the Rosaceae family, such as peach, plum, apricot, and sweet cherry, they occupy a pivotal position in the global temperate fruit industry. These fruits are highly valued by consumers for their flavor and nutritional attributes and have supported the development of high-value industrial chains in major production regions, thereby forming a cornerstone of local agricultural economies. Importantly, high yield and fruit quality are not determined solely during the ripening stage but largely depend on processes occurring during early fruit development.

Fruit early development, spanning from pollination, fertilization, and fruit set to the critical period of seed quality and pit hardening, represents a foundational determinant of reproductive success and yield formation in stone fruit species. This process typically follows a characteristic double sigmoid growth pattern and can be divided into three successive stages in *Prunus* species [1,2]. Stage I is characterized by rapid fruit growth driven mainly by intensive cell division and expansion, which are strongly promoted by auxin, GA, and CTK [3]. Stage II involves endocarp lignification and pit hardening, accompanied by relatively slow increases in fruit size, and is closely associated with changes in GA and ABA signaling [4]. Stage III is marked by rapid mesocarp enlargement and fruit maturation, during which ethylene plays an important regulatory role in fruit ripening and senescence. Stage I and Stage II include a series of highly coordinated cellular and molecular events, such as ovary expansion via cell division and enlargement and the formation of fruit tissues [5,6]. In contrast to later ripening processes that primarily modify fruit quality traits, this early fruit development is largely irreversible, as it determines whether the floral ovary successfully transitions into a viable fruit or undergoes fruit abortion, thereby directly linking reproductive efficiency to final yield [7,8]. Moreover, the ordered biological processes occurring during early fruit development predetermine final fruit size, shape, and seed vigor, thereby establishing the upper limit for yield potential and fruit quality. Any disruption in these processes, such as poor pollination and fertilization, premature fruit drop, or developmental arrest, would lead to a sharp decline in yield and significant economic losses for orchards. Therefore, a comprehensive understanding of the regulation mechanisms underlying early stone fruit development has significant importance for improving cultivation practices and breeding strategies.

Notably, early fruit development is initiated and coordinately regulated by a complex hormonal network. Within this network, auxin and GA act as core regulatory hormones, working alongside CTK, ABA, ethylene, and other important hormones to collectively modulate developmental signaling [9,10]. Auxin and GA synergistically promote ovary expansion, cell division, and fruit initiation and are widely recognized as the primary drivers of early fruit development across diverse species [11,12,13]. CTK further enhances early cell proliferation and directly impacts fruit size and pericarp development [14,15]. In contrast, ABA primarily functions as a regulator of developmental timing: it maintains ovary quiescence prior to pollination, whereas its downregulation following fertilization releases growth inhibition and permits fruit initiation [16,17]. In addition, ethylene mainly functions during fruit ripening and senescence, and its regulatory role in early developmental stages is relatively limited; therefore, it is not discussed as a primary focus in this review. Collectively, these hormonal signals establish the regulatory framework underlying fruit set, early growth, and yield potential, highlighting the importance of elucidating hormone interaction networks during early fruit development [18,19]. However, despite rapid progress in hormone biology, our understanding of how endogenous hormone networks are dynamically coordinated specifically during early stone fruit development remains fragmented and largely extrapolated from model plants. Therefore, this review systematically summarizes current research advances on the roles of endogenous hormones during early stone fruit development. It aims to deepen the theoretical understanding of the regulatory mechanisms governing stone fruit development and to provide a scientific reference for guiding production practices toward yield and quality improvement through hormonal regulation.

## 2. The Specific Roles and Dynamic Patterns of Key Endogenous Hormones

The early development of stone fruit is a sequential process regulated by multiple endogenous hormones. Through their specific sites of synthesis and dynamic patterns, these hormones collectively govern each critical event from fruit set to the end of pit hardening. This section will provide an in-depth analysis of the specific roles of key endogenous hormones during the early development process.

### 2.1. Auxin

Auxin, the first discovered plant hormone whose primary active form is indole-3-acetic acid (IAA), plays a central role in regulating plant cell expansion, tropic growth, and fruit development and ripening. Notably, auxin is especially critical during the initial phase of fruit development.

#### 2.1.1. Auxin Biosynthesis and Transportation

**Figure 1:**
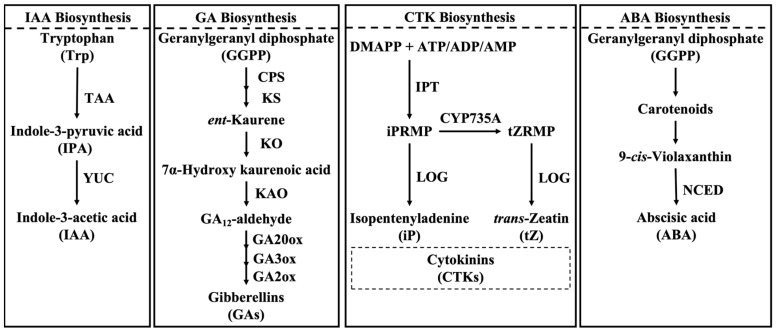
The main biosynthetic pathway of IAA, GA, CTK, and ABA in *Prunus* fruit. TAA, tryptophan aminotransferase of Arabidopsis; YUC, YUCCA, a flavin-containing monooxygenase; CPS, *ent*-copalyl diphosphate synthase; KS, *ent*-kaurene synthase; KO, *ent*-kaurene oxidase; KAO, *ent*-kaurenoic acid oxidase; GA20ox, GA20-oxidases; GA3ox, GA3-oxidases; GA2ox, GA2-oxidases; DMAPP, dimethylallyl diphosphate; IPT, isopentenyltransferase; iPRMP, isopentenyladenosine monophosphate; tZRMP, *trans*-zeatin riboside monophosphate; LOG, lonely guy; NCED, 9-*cis*-epoxycarotenoid dioxygenase.

The biosynthesis of auxin (primarily IAA) mainly relies on the tryptophan-dependent pathway (Figure 1). The most well-characterized and central route is a highly conserved two-step cascade [20]. In this pathway, tryptophan is first converted to the intermediate indole-3-pyruvic acid (IPA). This initial reaction is catalyzed by the TRYPTOPHAN AMINOTRANSFERASE OF ARABIDOPSIS (TAA) family of enzymes, with TAA1 being a key member whose activity is subject to precise post-translational regulation. For example, threonine 101 in the TAA1 protein could directly control its enzymatic activity, thereby regulating local auxin biosynthesis [21]. The second step is the conversion of IPA to IAA, which represents the rate-limiting step of the entire biosynthetic pathway. This reaction is catalyzed by YUCCA (YUC) family flavin monooxygenases, which oxidatively decarboxylate IPA, leading to the final production of IAA [22,23]. In stone fruit, key genes associated with this biosynthetic pathway have been progressively identified. During early fruit development in peach (*Prunus persica*), *PpTAA1* and *PpYUCCA2/4* genes were highly expressed specifically in the young embryo and ovary. Their peak expression coincided with the timing of a sharp increase in IAA content following fertilization [24], indicating that these genes played a predominant role in auxin synthesis during the initial phase of fruit development.

The asymmetric distribution of auxin within plant tissues is primarily attributed to its polar auxin transport (PAT). This process is characterized by strong directionality and is an active transport mechanism that depends on carrier proteins and energy expenditure, which serves as the fundamental basis for forming auxin concentration gradients [22]. The efflux of auxin is predominantly mediated by the PIN-FORMED (PIN) family of proteins, which serve as key efflux carriers. These proteins are asymmetrically localized on the plasma membrane, determining the direction of auxin efflux. The influx of auxin is primarily facilitated by the AUX1/LAX family of proteins, which are responsible for transporting auxin from the extracellular space or the cell wall apoplast into the cell interior [25,26,27]. In stone fruit species, the dynamic regulation of PAT is closely linked to the progression of fruit development. Research on sweet cherry (*Prunus avium* L.) showed that PAT activity was already maintained at a high level before flowering and was further enhanced after fertilization. Specifically, PAT activity reached the peak during the first three weeks post-fertilization, during which the quantity of endogenous IAA transported via the PAT pathway reaches its maximum [28]. This transport peak provides a crucial signal for the initiation of fruit development.

#### 2.1.2. The Pivotal Regulatory Role of Auxin

**Figure 2:**
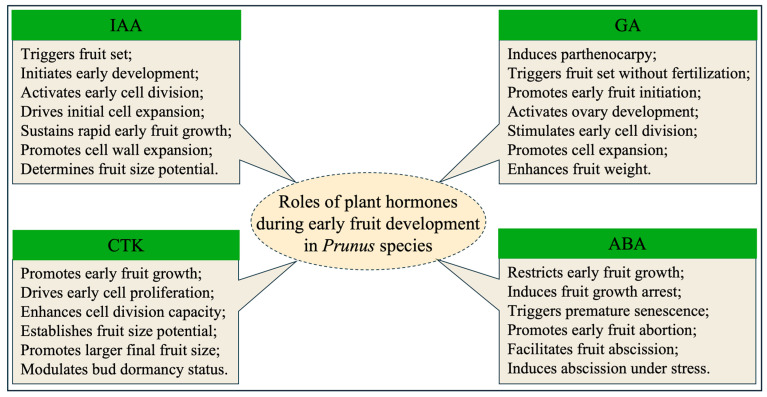
The regulatory role of IAA, GA, CTK, and ABA during early fruit development in *Prunus* species.

Auxin plays a central role in regulating fruit set and the initiation of early fruit development (Figure 2). In stone fruits, successful fruit initiation is highly dependent on both enhanced auxin biosynthesis and intensified polar auxin transport following fertilization. Auxin synthesis in the young embryo is rapidly activated after fertilization, and localized auxin production together with directional transport functions as a primary trigger for fruit set [29]. A pronounced increase in PAT following fertilization is therefore recognized as a critical developmental signal initiating early fruit growth. Once IAA concentration reaches a threshold level, auxin signaling transmits developmental cues to the young fruit, thereby activating programs for cell division and initial expansion [28]. Sustained auxin export from the ovary or young fruit to the maternal plant further acts as a signal indicating nutritional demand; disruption of this signal markedly increases the likelihood of fruit abscission. Experimental inhibition of PAT using TIBA (the PAT inhibitor) in sweet cherry pedicels significantly reduces auxin transport capacity, prevents effective IAA accumulation in young fruits, and consequently promotes fruit abscission, providing direct evidence for the essential role of auxin signaling in fruit set initiation [28].

Beyond fruit set, auxin also promotes cell division and expansion, thereby exerting a decisive influence on final fruit size (Figure 2). Serrani et al. (2007) confirmed that endogenous auxin directly drives early fruit growth by stimulating both cell division and expansion, which explain the consistently high auxin levels maintained in rapidly growing fruit tissues [30]. In stone fruits, this core mechanism has been well-documented. El-Sharkawy et al. (2014) compared two plum (*Prunus salicina* L.) cultivars with distinct patterns of fruit development and ripening, confirming that auxin served as a key hormonal stimulus for stone fruit development [31]. Moreover, auxin also coordinates the cytological development of the fruit by regulating downstream gene networks and deciding the final fruit size. At the cellular division level, a study has demonstrated a significant difference in auxin levels between normally developed fruits and aborted fruits in peach. Aborted fruits exhibited significantly lower IAA accumulation compared to normally developed fruits during the exponential growth phase, which was accompanied by the downregulated expression of genes related to DNA replication and the cell cycle, such as *PpCYCD2* and *PpCDKA* [32]. These findings indicate that sufficient auxin availability during early development is essential for sustaining active cell division. At the cellular expansion level, another study indicated that differences in fruit size between large-fruited and small-fruited apricots (*Prunus armeniaca* L.) were primarily determined by cell size [33]. During the cell expansion phase, the expression of auxin signaling transduction genes (such as *PaARF7* and *PaSAUR32*) and cell wall loosening-related genes (such as *PaEXP1* and *PaXTH2*) was significantly upregulated in large-fruited varieties. This suggests that auxin promotes cell water uptake and expansion by activating cell wall modification genes. In addition, Xu et al. (2018) demonstrated that changes in IAA content in different apricot cultivars showed a significant positive correlation with fruit growth indices including longitudinal and transverse diameters [34]. Compared to small-fruited cultivars, the IAA peak in large-fruited cultivars occurred earlier and was maintained for a longer duration, which indicated that the dynamic changes in IAA were highly synchronized with early fruit development.

Auxin additionally functions as a key survival signal through its polar transport in the pedicel, maintaining fruit attachment and suppressing early fruit drop. Through its polar transport in the fruit pedicel, auxin, as a survival signal, sustains the connection between the fruit and the tree, thereby suppressing early physiological fruit abscission. Research on sweet cherry confirmed that the PAT activity in the pedicels of young fruits about to abscise was at a low level. In contrast, PAT activity increased during critical stages of fruit development, such as the initiation of cell expansion [28,35]. These findings indicate that sustained and sufficient transport of IAA from the fruit to the maternal plant through the pedicel was necessary to inhibit abscission layer formation and maintain fruit attachment. In addition, the antagonistic interaction between auxin and ABA constitutes a key balancing mechanism in the regulation of early fruit drop. Zhou et al. (2024) demonstrated that the accumulation of auxin was reduced in aborted peach fruits, while ABA levels significantly increased, indicating that the attenuation of auxin signaling alongside the enhancement of ABA signaling during early development collectively triggered the cessation of fruit growth and the initiation of the abscission program [32].

### 2.2. Gibberellin

GAs are a class of diterpenoid phytohormones that play pivotal roles in flower bud differentiation, fruit set, early fruit development, and overall plant morphogenesis. Among the 136 known natural GAs, GA_1_, GA_3_, GA_4_, and GA_7_ are physiologically active. These active GAs have been widely used as plant growth regulators in agricultural production and play significant roles in promoting seed germination, stem elongation, fruit development, and enhancing plant stress resistance [36,37,38]. In stone fruits, GAs are particularly critical during early developmental stages, where they function as key growth-promoting signals supporting fruit initiation and subsequent enlargement.

#### 2.2.1. Gibberellin Biosynthesis and Transportation

GA biosynthesis is a multi-step pathway starting from geranylgeranyl pyrophosphate (GGPP) (Figure 1). GGPP is sequentially converted to *ent*-kaurene by CPS (copalyl pyrophosphate synthase) and KS (endogen-shell synthase), followed by oxidation steps that generate GA_12_-aldehyde and subsequently GA_12_, the common precursor of all gibberellins. GA_12_ is then converted in the cytoplasm into bioactive forms through the coordinated activities of GA20-oxidases (GA20ox) and GA3-oxidases (GA3ox), whereas GA2-oxidases (GA2ox) deactivate bioactive GAs and thereby fine-tune GA homeostasis [39].

GA signaling is primarily governed by the relief of growth repression mediated by DELLA proteins, a highly conserved derepression mechanism. DELLA proteins function as central repressors of GA responses by interacting with downstream transcription factors, such as PHYTOCHROME INTERACTING FACTOR 4 (PIF4), thereby blocking growth-related gene expression [40]. In the presence of GA, the hormone facilitated the interaction between the GID1 receptor (e.g., PsIGID1 in plum) and DELLA proteins, triggering DELLA ubiquitination and subsequent degradation. This process releases GA-mediated growth programs, including cell division and expansion [41,42]. In *Arabidopsis thaliana*, five DELLA proteins (RGA, GAI, RGL1, RGL2, and RGL3) function as core regulators that inhibit fruit initiation and growth [43]. In addition to the canonical DELLA-dependent pathway, GA signaling also operates through DELLA-independent mechanisms. Evidence indicates that even in the absence of DELLA proteins, GA can still promote fruit growth in specific tissues through a GID1 receptor–26S proteasome–SPATULA (SPT) transcription factor pathway, highlighting the complexity and flexibility of GA signaling during fruit development [44].

#### 2.2.2. The Pivotal Regulatory Role of Gibberellin

GA plays a central role in inducing parthenocarpy and promoting fruit set (Figure 2). In stone fruit, one of the most distinctive functions of GA is its ability to substitute for pollination and fertilization signals, thereby triggering fruit initiation in the absence of seed development. Exogenous application of GA_3_ to unpollinated pistils effectively induced fruit set and supports early fruit growth, demonstrating that GA acts as a potent signal for ovary activation [45]. In apricot, GA_3_ application at full bloom significantly increases fruit set rates and reduces early fruit drop, indicating that gibberellins promote ovary development and stabilize fruit retention during the critical post-anthesis period [46]. This indicated that gibberellin effectively promotes ovary development, prevents early fruit abscission, and ensures the successful initiation of fruit development.

Following fruit set, early fruit development largely depends on coordinated cell division and expansion, processes in which gibberellins play a key regulatory role (Figure 2). Elevated endogenous GA levels were associated with increased cell number and enhanced cell elongation, supporting rapid fruit growth during early developmental stages [36]. In plum (*Prunus salicina* L.), bioactive gibberellins such as GA_1_ and GA_4_ are essential for sustained fruit growth throughout ontogeny, and functional GA receptors, including PsIGID1, mediate GA-dependent degradation of DELLA repressors to activate downstream growth responses [31]. Additionally, this study also identified a functional gibberellin receptor (PsIGID1) in plum, which could form a complex with DELLA repressor proteins in a gibberellin-dependent manner, thereby relieving DELLA-mediated growth inhibition and activating downstream responses. This provided molecular evidence for the direct promotion of stone fruit cell growth by gibberellin. In peach fruit, young fruits treated with different concentrations of GA_3_ at 30 days after full bloom could promote fruit enlargement and increase single-fruit weight [47]. In sweet cherry, fruits treated with GA_3_ were significantly heavier than untreated fruits [48]. This evidence suggests that GA directly participated in the molecular programs driving fruit cell division and expansion, promoting the achievement of cell enlargement and nutrient accumulation. Beyond its direct effects on fruit set and early growth, GA also functions as an important regulator of endogenous hormone homeostasis. Integrated metabolomic and transcriptomic analyses in sweet cherry demonstrate that GA_3_ application not only elevates endogenous GA levels but also alters the metabolism of other hormones, including ABA and IAA. This hormonal reprogramming is accompanied by coordinated regulation of key biosynthetic genes, such as *NCED* and *YUCCA*, as well as GA-responsive transcription factors, including members of the WRKY family [49]. These findings indicate that GA-mediated regulation of early fruit development operates through an integrated transcriptional–hormonal network rather than through isolated signaling pathways. In addition, gibberellins play a critical role in breaking flower bud dormancy, thereby indirectly influencing fruit development. Successful fruit development in stone fruit depended on the ability of flower buds to overcome dormancy and achieve normal bloom. Insufficient winter chilling accumulation due to global warming could hinder dormancy release, thereby negatively impacting fruit set. Research has shown that the mechanism of certain agrochemicals used to promote dormancy release, such as hydrogen cyanamide and its alternatives, involves altering endogenous levels of GA [50]. Therefore, the regulation of GA not only directly influences fruit development after set but also indirectly determines the starting point of fruit development by affecting flowering quality.

### 2.3. Cytokinin

CTK is a chemical signal that plays a central role in plant development. Its functions are manifested in fundamental developmental processes, including the maintenance of shoot and root apical meristems, vascular differentiation, secondary growth, and floral initiation [51]. In fruit-bearing species, cytokinins are particularly important during early developmental stages, where they contribute to the establishment of fruit growth potential by controlling the intensity and duration of cell division.

#### 2.3.1. Cytokinin Biosynthesis and Transportation

CTK is primarily synthesized in the root tips and transported to the aerial parts via the xylem (Figure 1). However, the active cytokinins required for early fruit development do not rely exclusively on long-distance transport from roots, as developing fruits possess complete enzymatic machinery for cytokinin biosynthesis and activation [52,53]. The biosynthesis of major bioactive cytokinins, such as isopentenyladenine (iP) and *trans*-zeatin (tZ), occurs primarily within plastids [54]. In this process, dimethylallyl diphosphate (DMAPP) and AMP/ADP/ATP serve as substrates for isopentenyltransferase (IPT), which catalyzes the formation of cytokinin nucleotide precursors, such as isopentenyladenosine-5′-monophosphate (iPRMP). Subsequently, these inactive CTK nucleotide precursors are converted into active free-base forms through the action of phosphoribohydrolases encoded by the *LONELY GUY* (*LOG*) gene family. LOG enzymes directly dephosphorylate the inactive CTK nucleotide precursor iPRMP, converting it into the iP. In addition, CYP735A enzymes convert iP-type cytokinin precursors into *trans*-zeatin–type precursors, which are similarly activated by LOG-mediated dephosphorylation [53,55,56]. Cytokinin homeostasis is further regulated through degradation processes. Cytokinin oxidase (CKX) is a key enzyme that catalyzes the cleavage of the cytokinin side chain, leading to its permanent inactivation [53,57,58]. In peach, the expression levels of *CKX1* and *CKX2* increase during the later stages of early fruit development, suggesting that enhanced cytokinin degradation facilitates the transition from active cell division to cell expansion [59,60].

#### 2.3.2. The Pivotal Regulatory Role of Cytokinins

CTKs play a crucial role in regulating early fruit growth by promoting cell division (Figure 2). During the early post-fruit set stage in peach, particularly within the first 40 days after full bloom, the mesocarp undergoes intensive cell proliferation. This period coincides with a significant peak in the content of active cytokinins including tZ, iP, dihydrozeatin (DHZ), and their nucleosides. As fruit development progresses into later stages, active cytokinin levels decline sharply, consistent with reduced expression of cytokinin biosynthetic genes such as *LOG*. These coordinated changes indicate that cytokinins synthesized within the developing fruit act as pivotal regulators of early cell division and growth [59]. CTKs also exert a strong influence on final fruit size. In peach, large-fruit cultivars display significantly higher cytokinin levels and elevated expression of key biosynthetic genes, such as *PpIPT5a*, during the young fruit stage compared with small-fruit cultivars [61]. Similarly, in jujube, the cytokinin degradation gene *ZjCKX5* functions as a negative regulator of fruit size. Transcription factors ZjWRKY23 and ZjWRKY40 directly repress *ZjCKX5* expression, thereby enhancing cytokinin accumulation and promoting increased fruit size [62]. These findings collectively demonstrate that elevated cytokinin levels enhance cell division capacity, which directly contributes to greater final fruit volume. In addition to their role in fruit growth, cytokinins are involved in the regulation of bud dormancy. During dormancy maintenance, cytokinin biosynthesis is suppressed. In apricot, the DAM6 transcription factor represses cytokinin biosynthesis by downregulating the expression of *IPT* and *CYP735A* genes, while simultaneously upregulating the expression of *CKX* genes to accelerate cytokinin degradation. This coordinated regulation reduces cytokinin levels within buds and contributes to the maintenance of dormancy [54].

### 2.4. Abscisic Acid

ABA is not merely an abscission-related hormone during early stone fruit development but functions as a multifunctional signaling molecule involved in growth regulation, developmental timing, and stress adaptation. Through finely tuned networks of biosynthesis and signal transduction, ABA participates in key processes including fruit set determination, regulation of cell proliferation and expansion, initiation of ripening, and responses to environmental stress.

#### 2.4.1. Abscisic Acid Biosynthesis and Transportation

ABA is synthesized via the carotenoid cleavage pathway, also known as the indirect biosynthetic pathway (Figure 1). This biosynthetic process is initiated by the catalytic activity of 9-*cis*-epoxycarotenoid dioxygenases (NCEDs), a key enzyme family that catalyzes the rate-limiting step of ABA biosynthesis and has been extensively characterized in species such as tomato and grape [63,64]. In *Arabidopsis*, multiple *NCED* genes (*NCED2*, *NCED3*, *NCED5*, *NCED6*, and *NCED9*) collectively contribute to ABA biosynthesis [65]. In cotton, 23 *NCED* genes have been identified, with *GhNCED5*, *GhNCED6*, and *GhNCED13* showing strong correlations with ABA accumulation dynamics [66]. In addition, the immediate product of NCED-mediated cleavage, xanthoxin, is subsequently converted into ABA through the sequential action of short-chain dehydrogenase/reductase (SDR) and abscisic aldehyde oxidase (AAO), the latter requiring a molybdenum cofactor for activity [67]. ABA signal transduction operates through a conserved core module consisting of PYR/PYL/RCAR receptors, group A protein phosphatases 2C (PP2Cs), and SNF1-related protein kinase 2 (SnRK2) proteins [68]. In *Arabidopsis*, 14 PYR/PYL receptors, nine PP2Cs, and 10 SnRK2s have been identified [69,70]. Zeng et al. (2020) identified the core component-encoding genes from the peach genome, including seven *PpPYRs*, 10 *PpPP2Cs*, and seven *PpSnRK2s* [71]. Wang et al. (2015) identified three *PaPYLs*, six *PaPP2Cs* and six *PaSnRK2s* in sweet cherry. Among them, *PaPYL2/3*, *PaPP2C3/4/6* and *PaSnRK2/4* were highly expressed at the early stages of fruit development [72].

#### 2.4.2. The Pivotal Regulatory Role of Abscisic Acid

ABA is a dynamic regulator governing the entire fruit life cycle, from its early development and seed maturation to the initiation of ripening and senescence (Figure 2). In studies on peach, inter-specific hybridization has been shown to induce fruit abortion, resulting in growth inhibition during the exponential growth stage. This result indicated that ABA levels in aborted fruits were significantly higher than those in normally developing fruits. Elevated ABA concentrations suppressed cell growth by inducing the expression of the senescence-associated gene *PpeKIL1*, thereby ultimately blocking fruit development and triggering fruit abscission [32]. Wang et al. (2025) report that ABA played a key regulatory role in peach fruit abortion through transcription factor-mediated signal integration involving PpeSML1 [73]. Specifically, ABA directly activated the expression of fruit senescence-associated genes (*PpeBFN1* and *PpeBRN1*), thereby triggering an irreversible senescence program and ultimately promoting peach fruit abortion. This indicated that a central role of ABA during early fruit development was to act as a growth-inhibitory signal that triggered fruit growth arrest and even fruit abscission under specific conditions. Although the regulatory role of ABA during early stone fruit development remains less explored, Soto et al. (2013) demonstrated that exogenous ABA delayed ripening at mid-S3 but promoted ripening at later stages by modulating auxin- and cell-wall-related gene expression, highlighting the stage-dependent nature of ABA signaling [74]. Consistent with this functional complexity, recent evidence from peach ripening indicates that NAC transcription factors such as PpNAP4 directly activate ABA biosynthetic genes (*PpNCED2/3*), thereby linking hormonal regulation with metabolic reprogramming [75]. Beyond its inhibitory role in growth and ripening, ABA also functions as an early developmental cue with lasting structural consequences in stone fruits. In sweet cherry, ABA application at fruit set reprogrammed subsequent cell wall and cuticle development, resulting in enhanced deposition of covalently bound pectins and hemicelluloses and increased accumulation of long-chain aliphatic waxes at ripening. This structural reinforcement was associated with early activation of ABA biosynthesis (*PaNCED1*) and cuticle-related regulators (*PaWINA* and *PaLACS1*), indicating that ABA signaling at fruit set exerted a developmental pre-programming effect linking early hormonal signals to later fruit surface and cell wall maturation [76]. In addition, under stress conditions (drought, heat, cold and salinity) or failed pollination and fertilization, ABA could accumulate in the abscission zone of flower stalks or fruit pedicels, inducing programmed cell death and promoting abscission zone formation, ultimately leading to the abscission of flowers or young fruits [77].

## 3. The Interaction and Regulation of Endogenous Hormones

**Figure 3:**
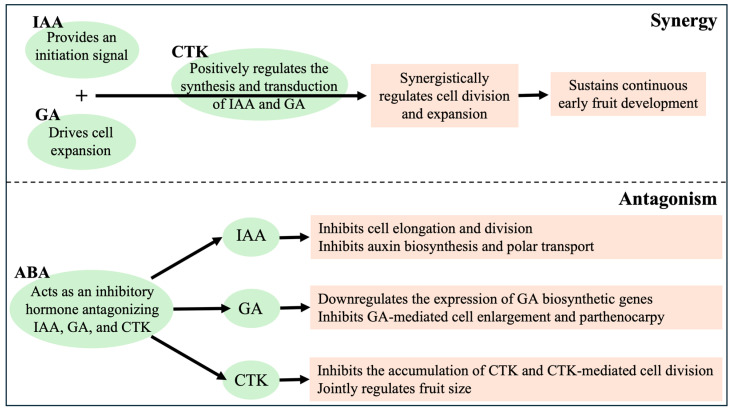
The interaction and regulation of IAA, GA, CTK, and ABA during early fruit development in *Prunus* species.

Early development of stone fruits is not regulated independently by a single hormone; rather, it is jointly mediated by IAA, GA, CTK, and ABA through complex networks of synergy, antagonism, and dynamic balance. Elucidation of the interactions among these hormones provides important theoretical support for understanding the regulatory mechanisms underlying early fruit development (Figure 3).

### 3.1. Synergistic Interactions Among Hormones

IAA, GA, and CTK act as major growth-promoting hormones and, through synergistic interactions, activate key programs of early fruit development, thereby driving fruit set as well as cell division and expansion. Accumulating evidence indicates that the synergistic interaction between IAA and GA constitutes a central regulatory module driving fruit set and parthenocarpy during early development of stone fruits. IAA and GA form a finely tuned regulatory network through the direct interaction between ARF/IAA transcription factor complexes and DELLA proteins, coordinating cell division and expansion to release the dormancy of ovary development [11,78]. In young peach fruit, exogenous spermidine transiently upregulated *CYCD3* and auxin biosynthesis genes (*TRPB*), supporting early cell division and expansion, highlighting its potential to reinforce the IAA–GA synergistic network during fruit set [79]. Both pollination-induced normal fruit set and pollination-independent parthenocarpy depend on the activation of this synergistic mechanism. At the molecular level, in the absence of hormonal signals, IAA proteins bind to ARFs to repress IAA-responsive genes, while DELLA proteins block the activation of GA downstream genes [80]. Following fertilization, IAA produced by developing ovules is perceived by TIR1/AFB receptors, triggering Aux/IAA degradation and releasing ARF activity [81,82]. Concurrently, IAA upregulates GA biosynthetic genes, such as *GA20ox* and *GA3ox* (e.g., *PpGA20ox1* and *PaGA3ox2* in peach), promoting the accumulation of bioactive GAs [49,83]. In *parthenocarpy*, combined IAA and GA treatments are more effective than either hormone applied alone [82]. In *Prunus* fruit species, increasing evidence supports the conserved role of auxin–GA synergism during early fruit development. In sweet cherry, Zhang and Whiting (2013) demonstrated that combined GA_3_ and auxin treatments improved fruit quality and development without impairing endocarp growth, highlighting the coordinated regulation of mesocarp expansion and pit formation [1]. In Japanese plum ‘Fengtangli’ (*Prunus salicina*), combined exogenous IAA and GA_3_ application significantly improved fruit set rate compared to single-hormone treatments, which was associated with enhanced expression of cell division genes (*CYCD3*) and cell expansion genes (*SAUR19*) [83]. In sweet cherry, exogenous GA_3_ treatment can directly induce parthenocarpy in sweet cherry, improving fruit set by regulating the synergistic effects among endogenous IAA and GA [49]. Mutant studies in *Arabidopsis thaliana,* tomato and fleshy fruits provided valuable mechanistic references for *Prunus* fruits [84,85,86], and similar synergistic mechanisms have been verified in peach and sweet cherry [2]. Overall, IAA provides the initiation signal, whereas GA supplies the driving force for cell expansion. The conserved nature of this synergistic mechanism provides a theoretical foundation for breeding improvement in stone fruits. CTK further reinforces fruit developmental signaling in stone fruits by forming a synergistic network with IAA and GA, with the core mechanism involving the positive regulation of IAA and GA biosynthesis and signal transduction by CTK [87,88]. CTK promotes IAA accumulation by inducing *YUC* family genes and suppressing IAA oxidases, while simultaneously upregulating GA biosynthetic genes. In turn, IAA and GA enhance the expression of CTK receptor genes, establishing a positive feedback loop that sustains early fruit growth [89].

### 3.2. Antagonistic Interactions Among Hormones

ABA acts as a core inhibitory phytohormone in plant development, forming extensive antagonistic relationships with IAA, GA, and CTK. The dynamic balance between ABA and these three growth-promoting hormones directly determines the fate of fruit development, including processes such as fruit set, cell expansion, ripening, and dormancy. The antagonism between ABA and IAA directly affects fruit cell elongation and parthenocarpic development. At the signaling level, the ABA receptor PYL8 interacts with MYB77 to enhance auxin-responsive gene expression; however, this crosstalk is tightly regulated by the ABA–IAA balance. Excessive ABA stabilizes Aux/IAA repressors and disrupts the TIR1/AFB–Aux/IAA–ARF signaling pathway, thereby inhibiting IAA-mediated cell elongation [20]. In peach, exogenous ABA downregulated the expression of auxin transport genes (*PpPINs*) and auxin receptor genes (*PpTIR1*), whereas exogenous auxin treatment upregulated these genes, thereby promoting cell elongation. Moreover, early fruit development requires high auxin (IAA) levels to stimulate ethylene production. ABA appears to modulate fruit development not only by interfering with ethylene biosynthesis and cell-wall-related genes but also by regulating auxin-associated signaling pathways [90]. In sweet cherry, WRKY transcription factors can bind to the promoters of hormone synthesis genes (e.g., *NCED*, *YUCCA*) to enhance the antagonistic effect of ABA and GA [49]. Physiologically, this antagonism is critical for fruit set and development: parthenocarpy cucumber genotypes maintain high endogenous IAA levels and low ABA levels during fruit development, whereas exogenous ABA suppresses fruit set that is otherwise rescued by combined IAA and zeatin treatment [91,92]. In addition, ABA inhibits auxin polar transport by regulating the localization and stability of PIN proteins, further restricting IAA-driven fruit expansion, and similar antagonism mechanisms have been verified in peach fruit, and similar antagonism mechanisms have been verified in peach fruit [90,93].

The antagonism between ABA and GA represents one of the best-characterized hormonal interactions and dominates the regulation of fruit ripening, seed dormancy, and postharvest behavior. ABA and GA mutually repressed each other’s biosynthesis: ABA could promote the expression of GA catabolic genes (e.g., *GA2ox*) and inhibit GA synthetic genes (e.g., *GA3ox*, *GA20ox*), while GA downregulated ABA biosynthetic genes (e.g., *NCED*) and activated ABA catabolic genes (e.g., *CYP707A*) [94,95]. At the signal level, DELLA proteins (core repressors of GA signaling) act as a key node connecting the two pathways—ABA enhances DELLA protein stability by inhibiting GA-induced ubiquitination degradation, thereby suppressing GA-mediated fruit cell expansion; conversely, GA promotes DELLA degradation to relieve its inhibitory effect on fruit growth [96,97]. In fruit development, this antagonism directly regulates fruit ripening and quality: ABA accumulation can promote chlorophyll degradation and sugar accumulation, while GA delays ripening by maintaining cell expansion potential [98,99].

CTK promotes fruit set and cell division, whereas ABA antagonizes these processes through opposing regulation of hormone biosynthesis and signaling, thereby influencing fruit size and yield. In sweet cherry, the exogenous GA_3_ significantly inhibited the synthesis of endogenous ABA in fruit by regulating the ABA synthesis genes [4,90]. Metabolically, ABA reduces CTK levels by repressing CTK biosynthetic genes (*IPT*, *LOG*) and enhancing CTK degradation via *CKX*, while CTK suppresses ABA biosynthesis by downregulating genes such as *ZEP* [100]. At the signaling level, ABA inhibits CTK-mediated cell division through SnRK2–ABI5 pathways, repressing CTK-responsive genes including *CYCD3* [101]. During fruit expansion, CTK promotes mesocarp cell division, whereas ABA restricts growth by inducing early senescence, jointly determining final fruit size [102]. Understanding the molecular mechanisms of these antagonistic interactions provides an important theoretical basis for agricultural practices such as regulating fruit set, improving fruit quality, and extending post-harvest shelf life.

### 3.3. Dynamic Regulatory Networks Among Hormones

Hormonal synergism and antagonism are not static but dynamically adjusted with developmental processes, tissue types, and environmental signals, forming a multi-dimensional regulatory network that ensures the orderly progression of plant developmental programs. During developmental progression, the balance between hormones shifts dramatically to meet stage-specific demands. In early embryogenesis, IAA and CTK exhibit antagonistic crosstalk at the root pole: elevated auxin levels upregulate CTK signaling repressors ARR7 and ARR15, attenuating CTK output and ensuring proper root pole specification [103]. In contrast, at the shoot apical meristem of post-embryonic plants, IAA promotes CTK responsiveness by repressing *ARR7/15* expression via ARF5/MP, reinforcing the CTK-mediated maintenance of stem cells [104]. Such stage-dependent switching of hormonal relationships ensures orderly phase transitions. Environmental signals act as external triggers to rewire hormonal networks. Drought stress enhances ABA accumulation, which antagonizes GA and CTK by stabilizing DELLAs and repressing auxin-responsive genes, thereby inhibiting growth and prioritizing stress tolerance [105,106]. Light conditions also modulate IAA–CTK–GA crosstalk: darkness promotes PIN protein degradation, reducing auxin transport, whereas CTK partially compensates for light deprivation to maintain shoot apical meristem activity [107,108]. These environmentally induced adjustments enable plants to coordinate growth with stress adaptation.

Dynamic crosstalk is anchored by core signaling hubs that integrate multiple hormonal pathways. DELLA proteins function as central nodes integrating ABA, ethylene, and auxin signals: ABA and ethylene stabilize DELLAs to suppress GA responses, whereas auxin promotes DELLA degradation to reinforce growth [109]. Similarly, the transcriptional corepressor TOPLESS integrates auxin, ABA, and jasmonate signaling by interacting with Aux/IAA proteins, AFPs, and JAZ–NINJA complexes, enabling context-dependent transcriptional regulation [110]. Together, these cascaded hubs form a robust multi-dimensional network, where hormonal synergism and antagonism are continuously recalibrated to ensure developmental programs proceed in an orderly and adaptive manner. This dynamic hormonal regulation is further reflected at the transcriptional level: high-resolution transcriptomic profiling of sweet cherry exocarp during development revealed finely tuned, stage-specific expression of over 34,000 contigs, including genes involved in sugar transport, cell wall remodeling, and cuticle formation [111]. In peach fruit, Torrigiani et al. (2012) found that polyamines can modulate such hormonal networks, inducing short-term auxin/GA-mediated growth and long-term ripening delay via coordinated regulation of auxin, GA, ABA, and ethylene-responsive genes [79]. These findings provide molecular evidence that endogenous hormone interactions orchestrate precise temporal and spatial control of early fruit growth and differentiation, linking hormonal signaling networks with downstream gene expression programs.

## 4. Conclusions and Perspectives

Early development is the most critical stage determining fruit set, yield potential, and final fruit quality in *Prunus* species fruit. This review emphasizes that the early developmental processes of *Prunus* fruits—including pollination, fertilization, fruit set, intensive cell division, and pit hardening—are tightly coordinated by an endogenous hormone network. Auxin, GA, CTK, and ABA constitute the core hormonal signals, and their dynamic balance dictates whether the ovary successfully develops into a fruit or undergoes growth arrest and abscission. Among them, auxin and GA function as the primary drivers initiating fruit set and promoting early growth, CTK reinforces cell proliferation and contributes to final fruit size, and ABA mainly serves as a negative regulator that restricts growth and promotes fruit abortion under unfavorable conditions.

Although the fundamental frameworks of hormone biosynthesis and signaling are largely conserved across plant species, stone fruits exhibit several distinctive features during early development. These include their strong dependence on successful fertilization and the rapid transition from intensive mesocarp cell division to endocarp lignification. Such characteristics make stone fruits particularly vulnerable to hormonal imbalance during early development, which partly explains the frequent occurrence of early fruit drop and unstable yields in many *Prunus* species. From a practical perspective, understanding endogenous hormone networks specifically in stone fruits is essential, rather than relying solely on knowledge from model plants or fleshy fruits lacking a hardened endocarp. Insights into hormonal regulation during early stone fruit development provide valuable guidance for orchard management. Rational application of plant growth regulators, optimization of fertilization strategies, and precise control of environmental conditions can be designed to favor growth-promoting hormonal signals while avoiding premature ABA accumulation. Such hormone-based regulation strategies may help stabilize fruit set, reduce early fruit drop, and improve yield consistency.

Future research on early stone fruit development should focus on several key directions to deepen mechanistic understanding and improve practical applications.

(i) Combining multi-omics analyses with accurate measurement of hormone levels and tissue-specific studies will be essential for understanding how hormone networks function in different parts of the fruit, especially the embryo, mesocarp, and endocarp. In addition, identifying key hormonal regulators associated with stable fruit set, reduced abortion, and optimized pit hardening will provide promising targets for molecular breeding and genome editing.
(ii) Understanding how hormonal networks respond to environmental stresses, such as drought, heat, and winter chilling, should be a key focus of future research. Studying how these stress-induced hormonal changes affect fruit development will help guide the development of stress-tolerant cultivars and more resilient orchard systems, which is increasingly important under climate change. Emerging technologies, including CRISPR-Cas9 for precise gene editing and high-throughput methods for measuring hormone levels, provide powerful tools to study these processes. These technologies could significantly advance the understanding of hormonal roles in fruit development, paving the way for more efficient breeding strategies that optimize fruit quality and yield.
(iii) Future studies could explore interdisciplinary collaborations with fields like microbiome, environmental science, and biotechnology. Integrating these areas could open new avenues for hormone-related research that enhances fruit quality, improves stress tolerance, and promotes sustainable agricultural practices. Ultimately, a deeper and more stone-fruit-focused understanding of endogenous hormone networks will not only advance fundamental knowledge of fruit developmental biology but also support the development of resilient, high-yield, and high-quality stone fruit production systems in the future. This knowledge could also help improve global stone fruit industries, particularly in regions impacted by climate change and soil health challenges, contributing to sustainable agricultural practices and food security.

Together, these research directions provide a systematic roadmap for advancing hormone-related research in stone fruit species and for bridging fundamental discoveries with practical applications.

## Data Availability

No new data were created or analyzed in this study.
